# Effects of multi‐electrode electrostatic precipitation during intraperitoneal aerosolized drug delivery: Insights from a large animal model

**DOI:** 10.1002/btm2.70142

**Published:** 2026-04-29

**Authors:** Mohammad Rahimi‐Gorji, Rosalie Ghesquière, Yiwen Long, Margo Vandenheede, Daryl K. A. Chia, Jesse Demuytere, Jana Zeebroek, Katleen Van Uytfanghe, Sarah Cosyns, Charlotte Debbaut, Wouter Willaert, Christophe Stove, Wim Ceelen

**Affiliations:** ^1^ Laboratory of Experimental Surgery (SURGX), Department of Human Structure and Repair Ghent University Ghent Belgium; ^2^ IBITECH‐BioMMedA, Department of Electronics and information systems Ghent University Ghent Belgium; ^3^ Cancer Research Institute Ghent (CRIG) Ghent Belgium; ^4^ Laboratory of Toxicology, Faculty of Pharmaceutical Sciences Ghent University Ghent Belgium; ^5^ Department of Surgery, National University Hospital National University Health System Singapore Singapore

**Keywords:** electrostatic precipitation, intraperitoneal aerosolized drug delivery, laparoscopic procedure, large animal experiment, peritoneal metastases

## Abstract

Intraperitoneal aerosolized drug delivery (IPADD) has emerged as a promising local treatment approach for peritoneal metastasis (PM). However, its effectiveness is currently limited by uneven aerosol distribution and restricted tumor tissue penetration. The use of electrostatic precipitation (ESP) exerts an electric force, which may enhance aerosol distribution and tissue penetration. A methylene blue (MB) solution was delivered intraperitoneally as an aerosol during laparoscopy in female pigs. Animals were randomized to receive either IPADD alone or IPADD combined with ESP. Tissue samples were taken from the ventral and lateral abdominal wall and from the small bowel. Aerosol distribution was evaluated using semiquantitative scoring of MB staining. Tissue penetration was measured on cryosections. Systemic resorption was assessed via serial blood sampling. Compared to IPADD alone, ESP clearly improved MB distribution, specifically in regions away from the nebulizer, and appreciably enhanced the tissue penetration in abdominal wall and small bowel samples. Sequenced activation of the three electrodes produced the most pronounced effects. ESP did not increase systemic exposure, and no tissue damage was observed. In this large animal model, multi‐electrode ESP safely enhanced aerosol distribution and tissue penetration during IPADD, showing potential to improve treatment in patients with PM.

## Graphical abstract

Electrostatic precipitation enhances intraperitoneal aerosolized drug delivery by improving spatial distribution and tissue penetration. In a large animal model, this approach achieved more uniform coverage and deeper uptake without increasing systemic exposure or causing tissue damage.

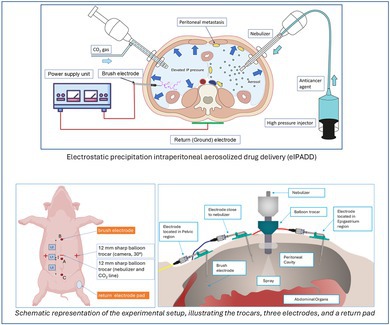

**Translational Impact Statement**

Intraperitoneal aerosolized drug delivery (IPADD) offers a minimally invasive treatment for peritoneal metastases but is limited by poor drug distribution and tissue penetration. This study demonstrates that electrostatic precipitation (ESP) enhances IPADD by improving aerosol uniformity and tissue uptake in a large animal model, without increasing systemic exposure. These findings support a clinically translatable strategy with the potential to increase chemotherapeutic efficacy and improve outcomes for patients with peritoneal cancers.

## 1 INTRODUCTION

Peritoneal metastasis (PM) is a devastating condition associated with a poor prognosis and severely diminished quality of life. Current treatment approaches typically rely on systemic chemotherapy with palliative intent, yet its efficacy remains limited due to inadequate blood supply to peritoneal metastases.^1^ Moreover, the peritoneal‐plasma barrier further restricts drug transport from the systemic circulation to the peritoneal compartment.^2^ This unique barrier, however, presents an opportunity for locoregional, intraperitoneal (IP) therapies to achieve high local drug concentrations with limited systemic toxicity.

Intraperitoneal drug delivery (IPDD) has emerged as an alternative approach to address the limitations of systemic chemotherapy. By delivering chemotherapeutic agents directly into the peritoneal cavity, IPDD circumvents the need for vascular drug transport, improving local drug exposure and minimizing systemic side effects.^3^ Techniques such as early postoperative IPDD and hyperthermic intraperitoneal chemotherapy (HIPEC) have demonstrated utility in well‐selected patients following maximal cytoreduction.^4^, ^5^ However, these approaches face challenges such as limited drug penetration, potential side effects, and occupational risks to healthcare workers.^6^, ^7^, ^8^

**FIGURE 1:**
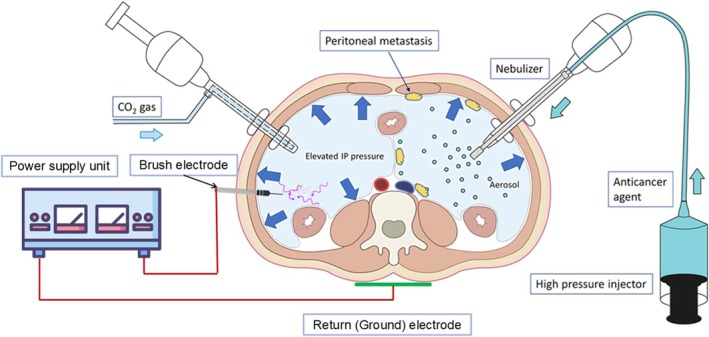
Electrostatic precipitation intraperitoneal aerosolized drug delivery (eIPADD). Adapted from Rahimi‐Gorji et al.^15^

To overcome these limitations, intraperitoneal aerosolized drug delivery (IPADD, also known as “pressurized intraperitoneal aerosol chemotherapy” or PIPAC) has been introduced.^9^ During IPADD, therapeutic compounds are nebulized intraperitoneally after establishing a CO_2_ capnoperitoneum (Figure 1).^10^ However, due to the effects of inertial motion, gravity, and anatomical properties of the peritoneal cavity, the spatial distribution of the IP aerosol is not homogeneous.^11^, ^12^ Electrostatic precipitation (ESP), originally developed for surgical smoke deposition during laparoscopy, may improve aerosol distribution^12^, ^13^ and enhance tissue penetration, as suggested by preclinical studies.^14^

**FIGURE 2:**
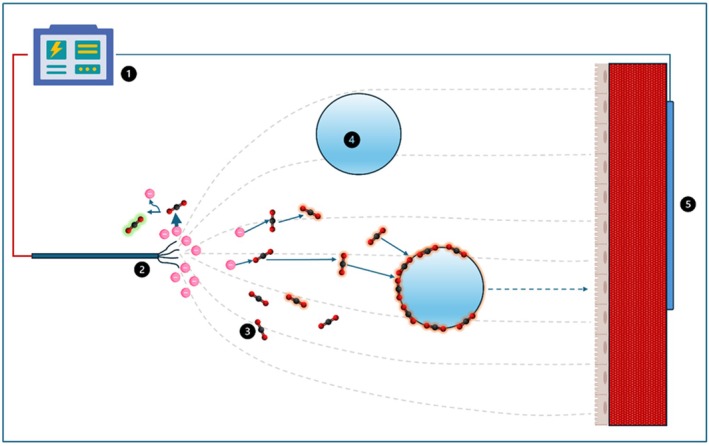
Mechanism of electrostatic precipitation during intraperitoneal aerosolized drug delivery. A high‐voltage generator (1) is connected to a stainless‐steel brush electrode (2), which serves as the cathode and is introduced into the peritoneal cavity. An adhesive return electrode (5), affixed externally to the abdominal wall, serves as the anode, completing the circuit. In the immediate vicinity of the electrode (2), a corona discharge is generated, leading to the ionization molecules (3). Close to the electrode, high‐energy electrons collide with CO₂ molecules, dislodging electrons and producing positively charged gas ions—indicated by a green glow. These positive ions remain near the cathode or are repelled depending on the local electric field. At greater distances from the electrode, where electrons move more slowly, CO₂ molecules instead absorb electrons and become negatively charged, represented by an orange glow. These negatively charged gas ions then transfer their charge to the suspended aerosolized drug droplets (4). The resulting negatively charged droplets are attracted across the electric field to the positively charged anode (5) (return electrode), where they deposit onto the abdominal wall. The dotted lines in the figure represent electric field lines guiding the movement of ions and droplets. The “green glow” and “orange glow” are included for visual reference only.

Electrostatic precipitation uses a high‐voltage generator (typically >6 kV) and a DC current to induce a corona discharge, ionizing and charging the aerosol droplets. The charged droplets are accelerated towards the abdominal wall and internal organs following electric current flux lines between a brush electrode and a grounding plate fixed to the patient's back or upper leg (Figure 2). Preclinical and clinical studies of ESP for smoke deposition have already demonstrated its safety.^16^

Despite these advancements, limited preclinical data exist on ESP‐enhanced IPADD (eIPADD) in large animal models.^13^, ^17^ In this study, the effects of ESP were examined on aerosol distribution, tissue penetration, local toxicity, side effects, and systemic resorption in a large animal model using a single or multiple electrode(s).

## 2 ANIMALS AND METHODS

### 2.1 Nebulized compound

Methylene blue (MB) was used as a model compound because its molar mass (319.85 g/mol) is similar to that of commonly used chemotherapeutics such as oxaliplatin (397 g/mol) and cisplatin (301.10 g/mol). More specifically, a commercially available MB solution (Sigma Aldrich, St. Louis, MO), quantified to contain 0.23 g MB/100 mL H_2_O (sect. 1 of Data S1, Supporting Information), was used as a surrogate for chemotherapy.

### 2.2 Animals

Female Landrace pigs (*n* = 15; RA‐SE Genetics, Ooigem, Belgium) were used with a weight ranging between 52 and 78 kg. Animals were acclimatized to their surroundings for a minimum of 7 days before the experiments and housed under standard conditions with unrestricted access to water and food. Daily evaluations for pain, discomfort, or signs of distress were conducted by the veterinarian of the animal facility. The experimental protocol was approved by the Animal Ethics Committee of the Faculty of Medicine and Health Sciences at Ghent University (ECD 24‐34), and experiments were conducted in compliance with relevant Belgian (Royal Decree of 29 May 2013) and European (Directive 2010/63/EU) animal welfare regulations.

### 2.3 Anesthesia

Pigs were fasted overnight and premedicated using intramuscular Sedaxyl™ (xylazine 2%) and Zoletil 100™ (tiletamine and zolazepam). Once adequately sedated, animals were transported to the operating room and intubated under general anesthesia with Esmeron™ (rocuronium), frocuronium, fentanyl, and sevoflurane inhalation. Animals received an ear vein cannula and a central venous line (right jugular). At the end of the experiments, animals were euthanized with T61™, administered via the jugular vein in accordance with approved protocols.

### 2.4 Experiments

#### 2.4.1 Intraperitoneal aerosolized drug delivery

All procedures were conducted in a controlled operating room environment (SURGX, Ghent University, Belgium), equipped with laminar airflow and maintained at a constant temperature of 20.5 ± 0.5°C to ensure optimal experimental conditions. A capnoperitoneum was created using an open approach, and three 12 mm balloon trocars were placed (Kii® Fios®, Applied Medical, Rancho Santa Margarita, CA). Intraperitoneal pressure was set at 10–15 mmHg using a pressure‐regulated insufflator (Olympus UHI‐4, Olympus Surgical Technologies Europe, Hamburg, Germany). This pressure range, which is also comparable to working parameters in laparoscopic surgery, was chosen to balance adequate visualization and working space with the need to minimize physiological compromise. In the context of trauma or penetrating injury models, higher insufflation pressures can elevate cardiovascular strain and increase the risk of barotrauma or tenting of the abdominal wall, which may lead to complications such as fascial tearing or compromised perfusion. A slightly lower pressure (range of 8–10 mmHg) was maintained in some cases due to the relatively small size of the abdominal cavity and to account for elevated baseline heart rate and blood pressure, thereby reducing animal stress and improving survival outcomes in line with ethical guidelines.

MB solution (170 mL) was nebulized using a HurriChem™ nebulizer (ThermaSolutions, White Bear Lake, MN) at a volumetric flow rate of 0.6 mL/s and upstream pressure of 21 bar (304 psi), in combination with a high‐pressure injector (Injektron 82M, Medtron AG, Saarbrücken, Germany). Following nebulization, the pneumoperitoneum was maintained in a steady‐state condition for 30 min. The peritoneal surfaces were imaged throughout using laparoscopic video imaging (EVIS EXERA III CV‐190, Olympus Surgical Technologies Europe, Hamburg, Germany).

#### 2.4.2 Electrostatic precipitation

**FIGURE 3:**
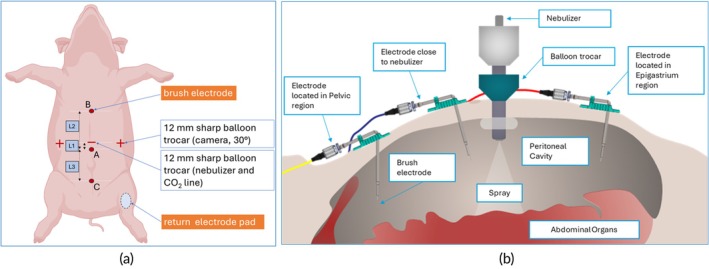
(a) Schematic representation of the experimental setup, illustrating the placement of three trocars, three brush electrodes, and a return pad (in the case of eIPADD). L1 (distance between electrode A and nebulizer) ≈35 mm, L2 (distance between electrodes A and B) ≈225 ± 25 mm, and L3 (distance between electrodes A and C) ≈145 ± 15 mm. (b) Relative positions of the nebulizer and brush electrodes.

A prototype power supply (Alesi Surgical, Cardiff, UK) was used for ESP, connected to three brush electrodes (IonWand™) and a return electrode was fixed to the animal's proximal lower limb or back (Figure 3a). The power supply featured three independent channels, each capable of generating voltages between 0 and 15 kV, with current intensities ranging from 10 to 100 μA. The electrodes were positioned at fixed distances on the midline: close to the nebulizer, in the epigastric region, and in the lower abdomen (Figure 3b).

The ESP system was activated at the start of nebulization and maintained for 30 or 60 min. Over the course of the experimental study, the ESP system was initially evaluated at increasing intensities and then adapted to improve deposition distribution. The experimental settings used in the six groups are detailed in Table 1.

**TABLE 1:** Overview of experimental groups and settings.

Group	Number of pigs	ESP setting (limits)			ESP duration (min)	Timing of laparotomy (after nebulization)	Notes
		A	B	C			
1	2	Not applicable			‐	30	No ESP
2	2	V_A_ = 10 kV I_A_ = 20 μA	V_B_ = 10 kV I_B_ = 20 μA	V_C_ = 10 kV I_C_ = 20 μA	30	30	3 electrodes‐Fixed
3	3	V_A_ = 15 kV I_A_ = 40 μA	V_B_ = 15 kV I_B_ = 40 μA	V_C_ = 15 kV I_C_ = 40 μA	30	30	3 electrodes‐Fixed
4	2	V_A_ = 15 kV I_A_ = 40 μA	V_B_ = 15 kV I_B_ = 40 μA	V_C_ = 10 kV I_C_ = 40 μA	30	30	3 electrodes‐Fixed
5	3	V_A_ = 15 kV I_A_ = 40 μA	V_B1_ = 15 kV V_B2_ = 5* kV I_B_ = 40 μA	V_C1_ = 5* kV V_C2_ = 15 kV I_C_ = 40 μA	30	30	*V_B_, V_C_ at 15 kV or reduced to as low as 5 kV, to match central electrode current. Alternating V_B_ & V_C_ settings. After 50% of volume is nebulized, with V_A_ maintained at a constant voltage
6	3	V_A_ = 15 kV I_A_ = 40 μA	V_B1_ = 15 kV V_B2_ = 5** kV I_B_ = 40 μA	V_C1_ = 5** kV V_C2_ = 15 kV I_C_ = 40 μA	P13: 30 P14 and P15: 60	P13: 30 P14 and P15: 60	**V_B_, V_C_ at 15 kV or reduced to as low as 5 kV, to match central electrode current. Sequencing V_B_ & V_C_ settings. Every 10s, with V_A_ maintained at a constant voltage

Abbreviations: A, central (umbilical) electrode; B, epigastric electrode; C, pelvic electrode; ESP, electrostatic precipitation; I, current; P, pig; V, voltage.

### 2.5 Experimental endpoints

#### 2.5.1 Aerosol distribution

Laparoscopic images were obtained before and 30 min after ESP activation, and additional images were taken after release of pneumoperitoneum and laparotomy. A midline laparotomy was performed after 30 min (after 60 min in two animals, Group 6) with the abdominal wall held up by stay sutures, ensuring that the ventral abdominal wall did not get in contact with the abdominal viscera. Detailed images were obtained from the following eight anatomical locations: left and right upper abdomen, left and right lower abdomen, dorsal region, ventral region, pelvic region, and epigastric region. Methylene blue staining of the peritoneal surface at each of these locations was quantified as follows: 0 = no staining; 1 = minimal staining; 2 = moderate staining; 3 = extensive staining. Scoring was done independently by two authors (MRG and WC) and average values were used for analysis.

#### 2.5.2 Tissue penetration

Standardized tissue samples were taken from both the peritoneal surfaces and the small bowel after laparotomy. Samples were embedded in clear disposable base molds using optimal cutting temperature (OCT) compound embedding medium (VWR International bvba, Leuven, Belgium) and snap‐frozen using dry ice at −20°C. Samples were cryosectioned into 100 μm slices using a cryostat (Leica Microsystems, Diegem, Belgium). Sections were mounted onto slides and fixed by immersing them in pre‐cooled acetone for 10 min. After air‐drying for a minimum of 2020 min, the OCT compound was removed by washing the slides in phosphate‐buffered saline (PBS). Each cryosection was then imaged under a light microscope (Leica Microsystems, Diegem, Belgium), and the images were imported into ImageJ software (National Institutes of Health, Bethesda, MD, available from https://imagej.nih.gov/ij/index.html) for further analysis of the penetration depth of MB. To analyze the images, the scale was calibrated using the 100 μm scale bar in each image for accurate pixel‐to‐distance conversion. MB intensity plots were generated to quantify penetration depth. Using the “Make Composite” function in ImageJ, the primary color channels (red, green, and blue) were separated for detailed analysis. Rectangular regions of interest (ROIs), oriented perpendicularly to the tissue surface, were delineated to assess the penetration profile. The intensity levels of the red and green channels were extracted through profile plots. These intensity values were averaged and inverted to create a distance‐versus‐intensity profile of the MB droplets, focusing on the blue color intensity data.

#### 2.5.3 Side effects

Potential adverse effects of the electrical currents on animal physiology were evaluated by continuous monitoring of respiratory rate, heart rate, electrocardiogram, and blood pressure during ESP. Additionally, animals were monitored for the occurrence of muscle twitching and other signs of interference with nerve and muscle conductivity.

#### 2.5.4 Tissue damage

Tissue injury was assessed at the end of each experiment via laparotomy, with the abdominal cavity fully opened and systematically examined. Two experienced surgeons evaluated all peritoneal surfaces for macroscopic signs of tissue damage, including burns, discoloration, or structural injury. This assessment was designed to capture immediate or early procedural effects. In addition, tissue samples (10 × 10 × 2 mm) were obtained from the peritoneum at four abdominal quadrants, and from the small bowel. Samples were fixed in 4% paraformaldehyde (PFA; VWR, Leuven, Belgium) in PBS for 72 h, dehydrated, and embedded in paraffin. Serial sections (4 μm thick) were prepared using a microtome (Microm HM355S, Thermo Scientific, Rockford, IL). H&E staining was performed using a LEICA ST5020 instrument (Leica Biosystems, Nussloch, Germany) following standard protocols. Slides were digitized using a whole‐slide imaging scanner (Pannoramic 250, 3DHistech, Hungary) and analyzed with 3DHistech's CaseViewer 2.3 software. Further analysis was conducted using QuPath, an open‐source whole‐slide imaging software suite.^18^ Peritoneal tissue sections were assessed for signs of architectural disruption or inflammation.

#### 2.5.5 Pharmacokinetics

Serial blood samples (5 mL) were collected from the first 13 pigs at 0, 15, 30, 35, 45, and 60 min post‐nebulization. For pigs 14 and 15 (Group 6), additional samples were collected at 65, 75, and 90 min. The 35‐min sample was excluded for pigs 14 and 15. Samples were immediately centrifuged and plasma was transferred to 1.5 mL tubes for quantitative analysis of MB. All samples were immediately stored at −20°C.

Quantitative analysis of MB in plasma was conducted using a liquid chromatography–tandem mass spectrometry method (LC–MS/MS) in which a Shimadzu Prominence LC‐system (Shimadzu, Kyoto, Japan) was coupled to an API 5500 triple quadrupole mass spectrometer (Sciex, Framingham, MA). Further details on the LC–MS/MS method are elaborated in sect. 2 of Data S1 (Figures S1 and S2 and Tables S1 and S2). Briefly, a standard addition approach was applied to quantify MB in the plasma samples. Each of the plasma samples collected at the different time points were split into 2 series of 5 aliquots of 45 μL, to which 5 μL of a spiking solution (or solvent control) was added. Four aliquots were used for spiking with MB, while a fifth aliquot served as a non‐spiked control by adding ultrapure water instead. All spikings were performed in duplicate (hence, the 2 series of 5 samples). Depending on the timepoint of sampling, spiking solutions at different MB concentrations were used: 1500, 3000, 6000 and 10,000 ng/mL were applied for the 0 and 15 min timepoints to increase the plasma concentration with 150, 300, 600, and 1000 ng/mL MB, respectively, while spiking solutions of 6000, 12,000, 24,000, and 40,000 ng/mL were used for the 30, 35, 45, 60, 65, 75, and 90 min timepoints, respectively, increasing the plasma MB concentration with 600, 1200, 2400, and 4000 ng/mL. All spiking solutions were prepared using certified reference MB powder (Sigma‐Aldrich, Diegem, Belgium), dissolved in methanol to obtain a working solution of 0.1 mg/mL. This working solution was then diluted with ultrapure water (produced using a Millipore purification system; Merck Millipore, Overijse, Belgium) to achieve the final required concentrations.

Sample preparation was based on a protocol published by Burhenne et al.^19^ After spiking, the samples were gently vortexed and equilibrated at 4°C for 1 h. Subsequently, 200 μL of 0.9% sodium chloride and 25 μL of internal standard (malachite green, 10 μg/mL in methanol) were added, followed by gentle vortexing and equilibration at 4°C for another 1 h. Next, 25 μL of calcium chloride solution (11.098 g/L dissolved in ultrapure water) and 1 mL of trifluoroacetic acid (10 g/L dissolved in acetonitrile) were added. The samples were then placed on a thermoshaker (TS‐100C, bioSan, Riga, Latvia) for 10 min at 23°C and 1400 rpm, followed by centrifugation for 10 min at 10°C and 3645*g* (Eppendorf 5804 R centrifuge, Hamburg, Germany). In the final step, 100 μL of the supernatant was added to 900 μL of diluent (0.1% formic acid +12.5% 50/50 (v/v) acetonitrile/methanol in ultrapure water) and 1 μL was injected onto the column. The performance of the analytical methodology was assessed via different means. To assess the precision of the analysis, samples were spiked in duplicate, with over 96% of the replicates showing no more than 15% deviation from their mean. Accuracy was evaluated using the samples spiked with 3000 ng/mL and 12,000 ng/mL respectively as a quality control sample. In over 93% of the cases, the pre‐set acceptance criterion of a 15% bias was met. Finally, at timepoint 0, the concentration of MB was required to be around 0 ng/mL as nothing was administered yet at that time. In absolute values, the largest absolute amount that was calculated for timepoint 0 was 71 ng/mL. However, calculating this relatively to the highest MB concentration obtained in the same pig, the ratio never exceeded 3.5% implying that the determined concentration for timepoint 0 resembles the measure uncertainty.

The LC–MS/MS data were analyzed using Sciex Analyst® 1.7.3 software. The determined concentrations were normalized based on the individual weight of each pig, relative to the average weight of all pigs. The area under the curve (AUC) was calculated using the trapezoidal rule for each individual pig to assess whether systemic absorption increases with the use of ESP. Based on a criterion applied in bio‐equivalence studies,^19^ a pre‐defined threshold of 25% above the AUC of reference pig 12 (P12, Group 1) was established to evaluate the difference.

### 2.6 Statistical analysis

Each experimental group consisted of two or three subjects. Data were visualized by plotting MB staining intensity as a function of distance, both with and without ESP, using the ggplot2 package in RStudio (licensed under the GNU Affero General Public License v3). To capture trends in staining intensity across distance, fitted curves were generated using LOESS (locally estimated scatterplot smoothing), a nonparametric method that models local relationships within the data.

## 3 RESULTS

### 3.1 Effects of ESP on aerosol droplet distribution

**FIGURE 4:**
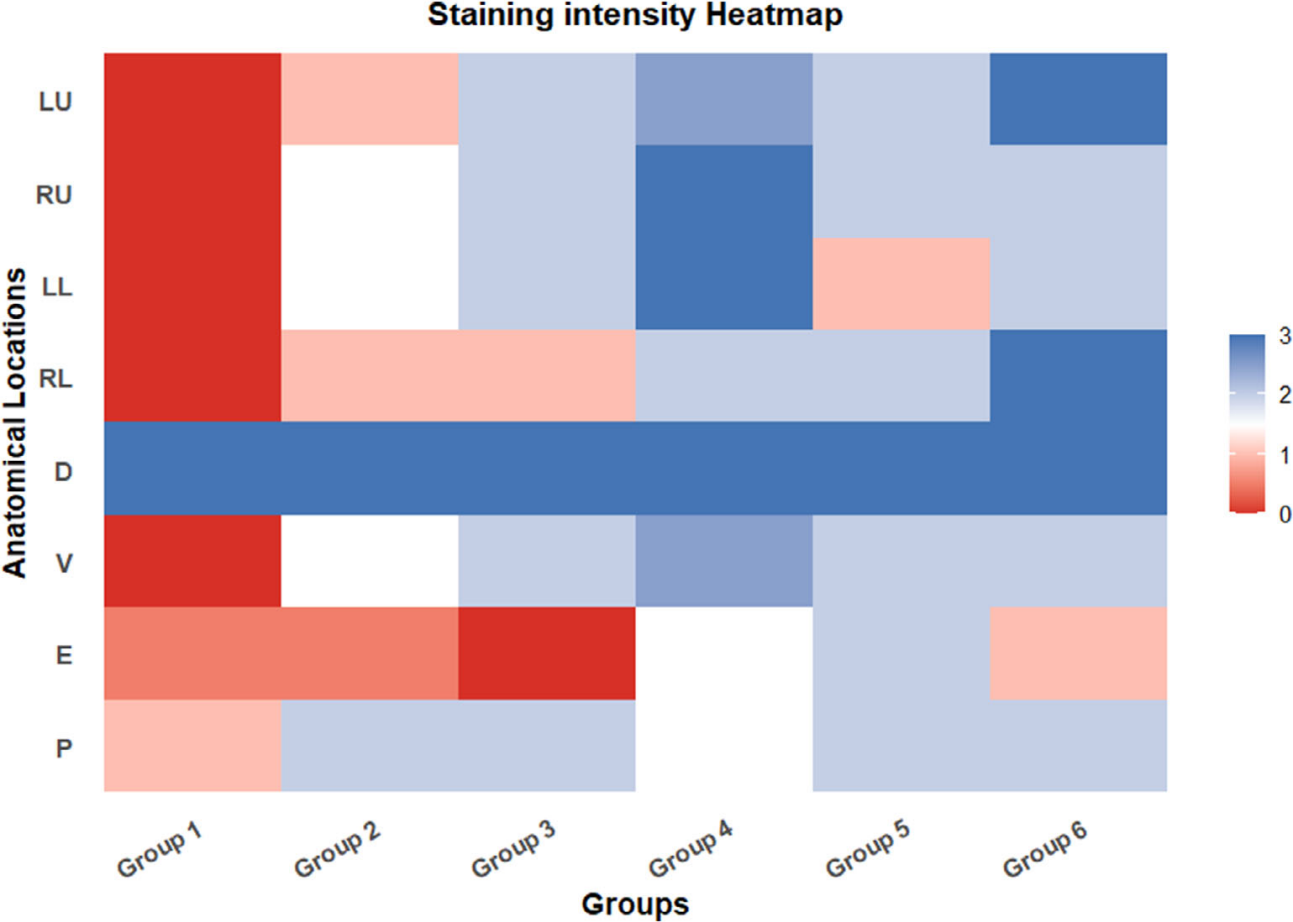
A heatmap of methylene blue staining scores at different anatomical locations. Scoring was done as follows: 0 = no staining, 1 = minimal, 2 = moderate, and 3 = extensive staining. LU, left upper abdomen; RU, right upper abdomen; LL, left lower abdomen; RL, right lower abdomen; D, dorsal region; V, ventral region; E, epigastric region; P, pelvic region. The ESP settings of the different groups are detailed in Table 1. Group 1 = no ESP, Groups 2–6 = ESP with three electrodes.

**FIGURE 5:**
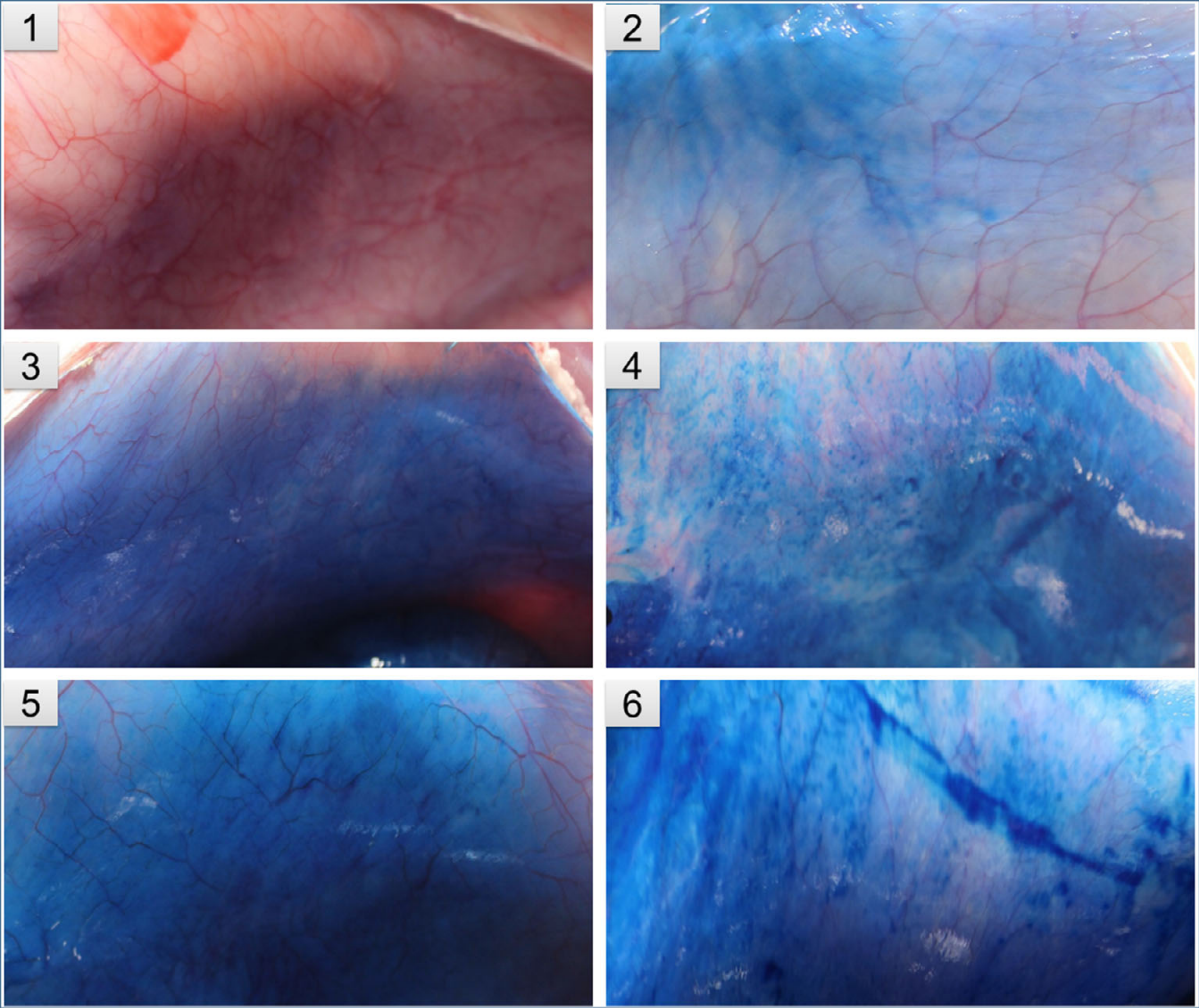
Representative staining patterns of the exposed ventral abdominal wall surface in the six experimental groups after methylene blue nebulization. Treatment groups are identified in Table 1.

The results of the semiquantitative scoring of MB coverage are shown in Figure 4. Clearly, the addition of ESP increased MB coverage, with maximal effects seen in Groups 5 and 6. As expected, the dorsal region, opposite the nebulizer nozzle, was maximally exposed, with or without ESP. Representative images of the stained ventral abdominal wall are shown in Figure 5.

### 3.2 Tissue penetration depth

**FIGURE 6:**
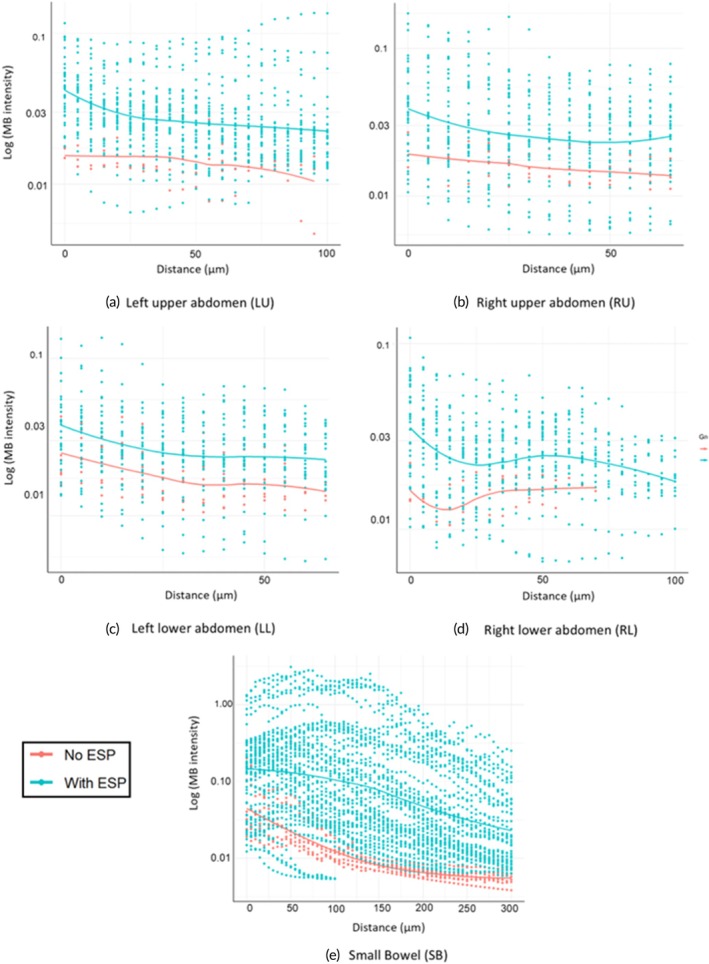
Methylene blue (MB) penetration in peritoneal tissue samples without and with ESP. The vertical axis represents the logarithmic values of MB intensity. Dots indicate MB intensity measurements from different regions of interest within the tissue samples, while lines represent the fitted curve based on LOESS (locally estimated scatterplot smoothing). The plots were generated using the ggplot2 package in RStudio under the GNU Affero General Public License v3.

**FIGURE 7:**
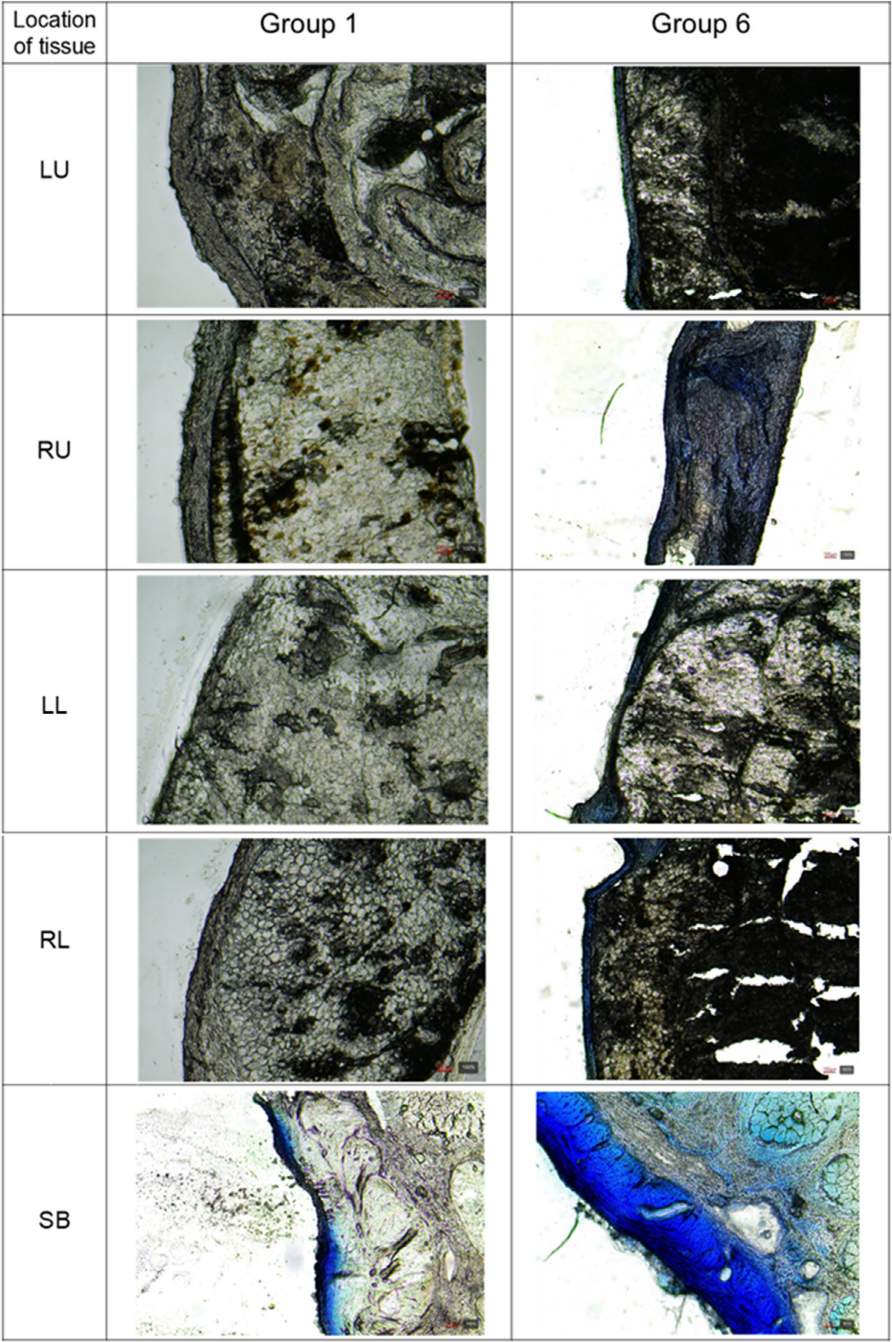
Representative cryosections of peritoneal tissue samples following aerosolized methylene blue administration, comparing aerosolization without ESP (Group 1) and with ESP (Group 6). Five tissue samples were collected from different regions of the peritoneal cavity: Left upper (LU), right upper (RU), left lower (LL), right lower (RL), and small bowel (SB). In each panel, the exposed tissue surface is oriented to the left. All sections were stained to visualize methylene blue penetration, with intensity and depth of blue coloration indicating dye distribution within the tissue layers. ESP application resulted in visibly deeper and more uniform tissue penetration compared to aerosolization without ESP. Scale bar = 100 μm for all images.

To quantify MB penetration depth, distance‐intensity plots were generated based on the analysis of cryosectioned stained tissue samples (Figure 6). Measuring droplet penetration depth in the lateral abdominal wall samples (LU: left upper; RU: right upper; LL: left lower; RL: right lower) was challenging due to low MB staining intensity. However, as shown in the plots, ESP application led to a considerable increase in MB intensity values in these regions. In contrast, small bowel (SB) tissue samples demonstrated a more pronounced enhancement in penetration depth with ESP. The intensity plots clearly indicate that ESP facilitated deeper tissue penetration, an effect that became even more pronounced when adjusting ESP technical parameters, such as sequencing the current between electrodes B and C. Representative histological images are shown in Figure 7.

### 3.3 Local toxicity

**FIGURE 8:**
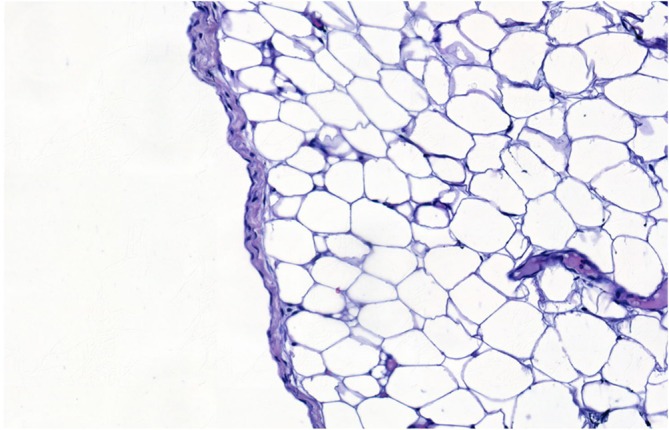
Representative image of H&E‐stained abdominal wall porcine peritoneum, harvested from the left lower abdominal quadrant. A mesothelial monolayer (indicated by an arrow) covers a thin layer of submesothelial stroma (indicated by an asterisk (*),(*), supported by adipose tissue (indicated by a cross (**†**)). No signs of inflammatory cell infiltration or tissue damage were observed after adding ESP to the platform. Magnification: ×100.

H&E‐stained tissue sections of the abdominal wall peritoneum across four quadrants did not reveal signs of disrupted tissue architecture. The mesothelial monolayer remained intact, covering a thin submesothelial stroma, with no evident inflammatory cell influx in all experimental conditions. A representative image of a section is shown in Figure 8.

### 3.4 Pharmacokinetics

**FIGURE 9:**
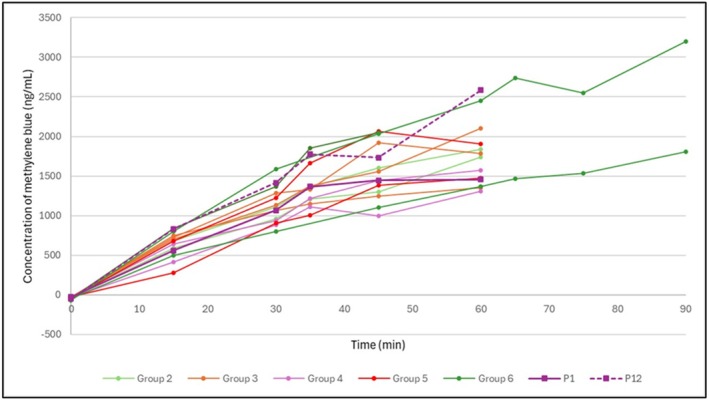
Time versus Methylene blue concentration with pig 12 (P12) (no electrostatic precipitation) indicated as reference animal. Treatment groups are identified in Table 1.

The plasma MB concentrations over time are shown in Figure 9. Animal P12 is used as the reference pig (without ESP, Group 1). Although Pig 1 also belonged to Group 1 and did not undergo ESP, it was not used as a reference due to the surgery being performed using a lower intraperitoneal pressure (which was necessary to maintain hemodynamic stability under anesthesia). A time‐dependent increase in MB concentrations was observed in all animals. Comparison of the AUC values for the different animals relative to the reference animal showed that the addition of ESP did not result in enhanced systemic absorption (sect. 3 of Data S1, Figure S3, and Table S3). Indeed, in none of the tested conditions was the pre‐defined threshold of 25% above the AUC of the reference animal used as the cut‐off point. The highest AUC observed, recorded in a Group 6 pig, was only 4% above that of the reference. Two animals were excluded from the AUC analysis due to missing data: Pig 11 (Group 5) lacked a weight measurement, and timepoint 60 was not collected for Pig 13 (Group 6).

## 4 DISCUSSION

IPADD has emerged as a promising minimally invasive surgical approach for the treatment of PM.^20^ Given that PM typically disseminates widely throughout the peritoneal cavity, achieving a uniform spatial distribution of aerosolized anticancer agents is critical for effective therapy. However, current aerosol technologies used in clinical settings produce relatively large droplets with high initial velocity, which often leads to suboptimal distribution due to inertial impaction and gravitational settling. This uneven deposition limits drug exposure in essential anatomical regions and may compromise overall therapeutic efficacy.

In this study, using MB as a model compound, we assessed the performance of a novel multi‐electrode ESP system by comparing drug distribution, tissue uptake, and systemic absorption following standard IPADD and eIPADD. Utilizing a large animal model, which closely resembles human anatomy and physiology, we demonstrated that the application of an electrostatic field during IPADD considerably improved the homogeneity of aerosol distribution across the peritoneal cavity and increased tissue penetration depth without adversely affecting systemic absorption as assessed by quantitative analysis of MB plasma concentrations.

The goal of the ESP system was to achieve effective aerosol deposition without requiring the surgical team to make adjustments based on subject or aerosol conditions. Electrode currents were typically below 20 μA at 15 kV, provided the electrode brush was kept sufficiently distant from nearby tissue. Symmetrical placement along the subject's midline was also necessary to minimize lateral variation in deposition.

Aerosol deposition was highest on ventral surfaces within 5 cm of the electrode catheter, and similarly at other points within that range. With voltage and current limits set to 15 kV and 40 μA, ESP was highly effective, though uniform distribution across the entire cavity was not always achievable. Good deposition could be obtained either in the pelvic and ventral regions or in the ventral and epigastric regions, but not both simultaneously. To address this, deposition was split into phases, with ESP deactivated alternately in either the pelvic or epigastric regions. In Group 5, this was done manually based on expected aerosol injection time. In Group 6, this alternating deactivation was automated in the ESP control system, enabling more frequent switching without requiring prior knowledge of injection duration.

Based on published data, the aerosol droplets were mostly in the 20–30 μm range. It became evident that the position of the central electrode relative to the nebulizer was critical to extending droplet flight time, allowing better interaction with the electric fields of distant electrodes.^21^, ^22^

In the absence of electrostatic assistance, aerosol distribution was predominantly influenced by gravitational force, resulting in very limited deposition on the side walls, ventral surface, epigastric region, and pelvic area. Staining was largely confined to the dorsal region, opposite the nebulizer, indicating that gravitational settling was the primary mechanism of droplet dispersion. These findings are consistent with prior studies highlighting the limitations of passive aerosol delivery, in which droplets preferentially accumulate in dependent regions rather than achieving a uniform spatial spread.^12^

When the ESP system was applied, a notable increase in the percentage‐stained surface area was observed, particularly on the side walls and ventral abdominal wall. The effect was most pronounced as the ESP voltage and current increased, suggesting that higher electrostatic forces enhanced aerosol attraction and adherence. Sequencing voltage and current between electrodes B and C further improved homogeneity, leading to broader aerosol coverage and more even deposition across internal surfaces. Among the tested configurations, Group 6 achieved the most uniform aerosol distribution, reinforcing the hypothesis that alternating electrostatic fields play an active role in optimizing drug deposition. The electrostatic forces counteracted gravitational and inertial effects, allowing droplets to reach areas that typically receive inadequate coverage. Despite these improvements, deposition remained somewhat limited in the sub‐diaphragmatic and pelvic regions, suggesting potential limitations in electrostatic influence at greater distances from the nebulizer. Notably, the pelvic area is spatially constrained, increasing the likelihood of positioning the electrode too close to pelvic organs and surfaces.

These findings underscore the effectiveness of ESP in improving aerosol homogeneity, which could help address inconsistencies in drug coverage seen with conventional IPADD. Additionally, ESP‐enhanced aerosol delivery could be particularly beneficial in clinical scenarios where uniform drug exposure is critical.

In addition to enhancing aerosol distribution, ESP increased tissue penetration depth, particularly in the side walls (LU, RU, LL, and RL) and small bowel (SB) regions. This effect became even more pronounced in Groups 5–6, where sequencing ESP settings were applied. The deeper penetration observed in samples of small bowel suggests that electrostatic forces facilitated droplet diffusion, overcoming diffusive resistance and interstitial fluid pressure that typically hinder drug penetration.

Tissue composition also played a key role in penetration depth. In abdominal wall tissues, a thin peritoneal membrane (~50–100 μm) is supported by a substantial fat layer, which acts as a barrier to deeper drug diffusion. Fat‐rich regions exhibited limited MB penetration, even with ESP, indicating that anatomical constraints may reduce the effectiveness of electrostatic‐assisted delivery in certain tissues. In contrast, small bowel tissue, which has a lower fat content and a more absorptive structure, demonstrated greater MB penetration, further supporting the role of ESP‐enhanced diffusion in facilitating deeper tissue uptake. These results highlight the potential clinical advantages of ESP in improving drug penetration, particularly in regions with less fat obstruction. While side wall penetration remains limited, the observed differences suggest that ESP‐enhanced IPADD could be optimized for targeted peritoneal therapies, ensuring that aerosolized drugs reach deeper layers where tumor nodules may reside.

A critical aspect of this study was to assess whether ESP induces any adverse physiological effects. Histological evaluation using H&E staining revealed no signs of inflammatory cell infiltration, necrosis, or structural tissue damage. These findings indicate that ESP‐assisted aerosol delivery does not compromise tissue viability and is unlikely to trigger adverse inflammatory responses. The lack of observable histological damage suggests that the electric field strength and exposure duration used in this study were within safe physiological limits, supporting the feasibility of ESP‐based drug delivery for clinical applications.

Because systemic drug resorption may be an important cause of systemic adverse events, we additionally assessed to what extent MB was present in the systemic circulation following the different treatments. More specifically, by quantifying MB at different time points post drug delivery, an AUC could be derived, allowing objective comparative analysis between the different drug delivery strategies. Our results indicated that none of the animals exceeded the pre‐defined threshold of 25% above the AUC of the reference animal (P12). These findings further support the potential of eIPADD, as the improved spatial distribution and tissue penetration of MB was not associated with an increased systemic absorption.

**Several limitations should be mentioned.** First, MB was used as a non‐toxic surrogate to evaluate aerosol behavior, deposition, and tissue distribution. While MB has a molar mass similar to commonly used chemotherapeutic agents (oxaliplatin, cisplatin, mitomycin C), it does not fully replicate properties such as charge, lipophilicity, or tissue binding. Therefore, tissue penetration observed with MB may differ from that of actual chemotherapeutics, especially higher molecular weight compounds such as paclitaxel or doxorubicin. Also, given the limited possibilities to induce peritoneal metastases in pigs, we did not study drug transport in tumor tissue, which is hindered by the adverse biophysical properties of the TME. However, the focus of this study was mainly on systemic absorption, since recent clinical reports suggest that ESP may increase plasma exposure of aerosolized drug, a finding we could not replicate in the pig model. Despite these limitations, this study provides foundational data on the combination of intraperitoneal aerosolized drug delivery and electrostatic precipitation, which can guide future experiments in tumor‐bearing models with clinically relevant chemotherapeutic agents.

To conclude, using MB as a model compound, this study provides strong experimental evidence that ESP meaningfully improves spatial aerosol distribution and enhances tissue penetration depth without inducing tissue damage or increased systemic absorption. Our findings suggest that ESP‐assisted intraperitoneal drug delivery could offer a major advantage over conventional IPADD/PIPAC by addressing uneven drug distribution and enhancing peritoneal tissue uptake. When validated in human studies, ESP has the potential to enhance the efficacy of intraperitoneal drug delivery.

## AUTHOR CONTRIBUTIONS

**Mohammad Rahimi‐Gorji**: Writing – review & editing; writing – original draft; visualization; methodology; investigation; data curation; conceptualization. **Rosalie Ghesquière**: Writing – review & editing; writing – original draft; visualization; methodology; investigation; data curation. **Yiwen Long**: Writing – original draft; formal analysis; data curation. **Margo Vandenheede**: Writing – original draft; data curation; methodology. **Daryl K. A. Chia**: Writing – original draft; data curation; methodology. **Jesse Demuytere**: Writing – original draft; data curation; methodology. **Jana Zeebroek**: Data curation. **Katleen Van Uytfanghe**: Writing – review & editing; methodology; investigation. **Sarah Cosyns**: Writing – original draft; data curation. **Charlotte Debbaut**: Writing – review & editing; supervision; methodology. **Wouter Willaert**: Writing – review & editing; methodology. **Christophe Stove**: Writing – review & editing; supervision; methodology. **Wim Ceelen**: Writing – review & editing; supervision; methodology; funding acquisition; conceptualization.

## CONFLICT OF INTEREST STATEMENT

The authors declare no conflicts of interest.

## ETHICS STATEMENT

This study was approved by the Medical Ethics Committee of Ghent University Hospital (UZ Ghent) under approval number ECD 24‐34 and was conducted in compliance with the committee's ethical standards. Animal handling and care adhered to both national and institutional guidelines.

## Supporting information


**Figure S1.** Gradient used for separation of compounds in the liquid chromatography.
**Figure S2.** Example of a chromatogram of the 45‐min timepoint spiked with 4000 ng/mL. The blue peak represents the quantifier ion of methylene blue, the red peak the qualifier ion of methylene blue, the light green peak represents the quantifier ion of malachite green, and the dark green peak the qualifier ion of malachite green.
**Table S1.** Retention time (RT), multiple reaction monitoring transitions and compound‐specific mass spectrometer settings. DP, declustering potential; EP, entrance potential; CE, collision energy; CXP, collision cell exit potential.
**Table S2.** System‐specific parameters of the mass spectrometer.
**Figure S3.** Illustration of applying the standard addition method to quantify the concentration of methylene blue present in a sample.
**Table S3.** Absolute values of area under the curve (AUC) (corrected for individual weight) for each individual pig and corresponding values expressed relative to the AUC of reference pig 12 (P12).

## Data Availability

The data that support the findings of this study are available on request from the corresponding author. The data are not publicly available due to privacy or ethical restrictions.
