# Supplementary material for: Effects of multi‐electrode electrostatic precipitation during intraperitoneal aerosolized drug delivery: Insights from a large animal model
Source: Bioeng Transl Med. 2026 Apr 29;11(4):e70142. doi: 10.1002/btm2.70142 (PMC13327604; doi:10.1002/btm2.70142)
Supplement: Supplementary file 1 — Figure S1. Gradient used for separation of compounds in the liquid chromatography. Figure S2. Example of a chromatogram of the 45‐min timepoint spiked with 4000 ng/mL. The blue peak represents the quantifier ion of methylene blue, the red peak the qualifier ion of methylene blue, the light green peak represents the quantifier ion of malachite green, and the dark green peak the qualifier ion of malachite green. Table S1. Retention time (RT), multiple reaction monitoring transitions and compound‐specific mass spectrometer settings. DP, declustering potential; EP, entrance potential; CE, collision energy; CXP, collision cell exit potential. Table S2. System‐specific parameters of the mass spectrometer. Figure S3. Illustration of applying the standard addition method to quantify the concentration of methylene blue present in a sample. Table S3. Absolute values of area under the curve (AUC) (corrected for individual weight) for each individual pig and corresponding values expressed relative to the AUC of reference pig 12 (P12). [file BTM2-11-e70142-s001.docx]

SUPPLEMENTARY DATA

1. Quantification of the commercially available methylene blue solution

The concentration of the methylene blue (MB) solution used during the animal experiments was determined by comparing it to a certified reference powder of MB. Starting from the reference powder, a standard solution was made with a precisely known concentration (based on the Certificate of Analysis), which was then diluted following a serial dilution protocol to a working solution. The same dilution protocol was applied to the commercially available MB solution. By back-calculating compared to the known concentration of the reference standard, the concentration of the MB solution was determined to be 0.23 mg/100 mL.

1. The liquid chromatography-tandem mass spectrometry method
   1. Liquid chromatography

Chromatographic separation was performed using a Phenomenex Kinetex 2.6 µm C18 100 Å ( 50 x 3.0 mm) column equipped with a UPHLC C18 guard column (Phenomenex, Torrance, CA, USA), maintained at 55 °C. The mobile phases consisted of 5 mM ammonium formate and 0.05% formic acid in ultrapore water (A) and 0.05% formic acid in 50/50 (v/v) acetonitrile/methanol (B). Separation was achieved using gradient elution at a flow rate of 0.40 mL/min with a total run time of 8 min (Suppl. Fig.1). The autosampler temperature was maintained at 4 °C.


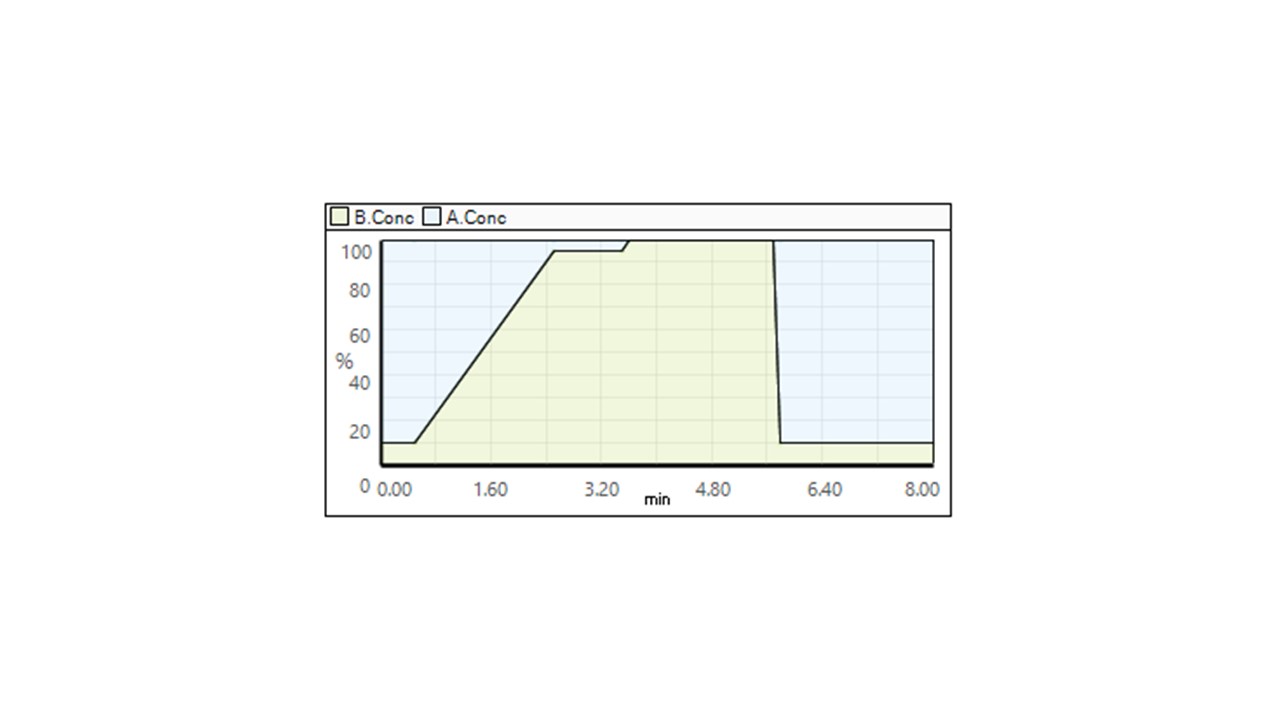


*Supplemental Figure 1****:*** *Gradient used for separation of compounds in the liquid chromatography.*

*
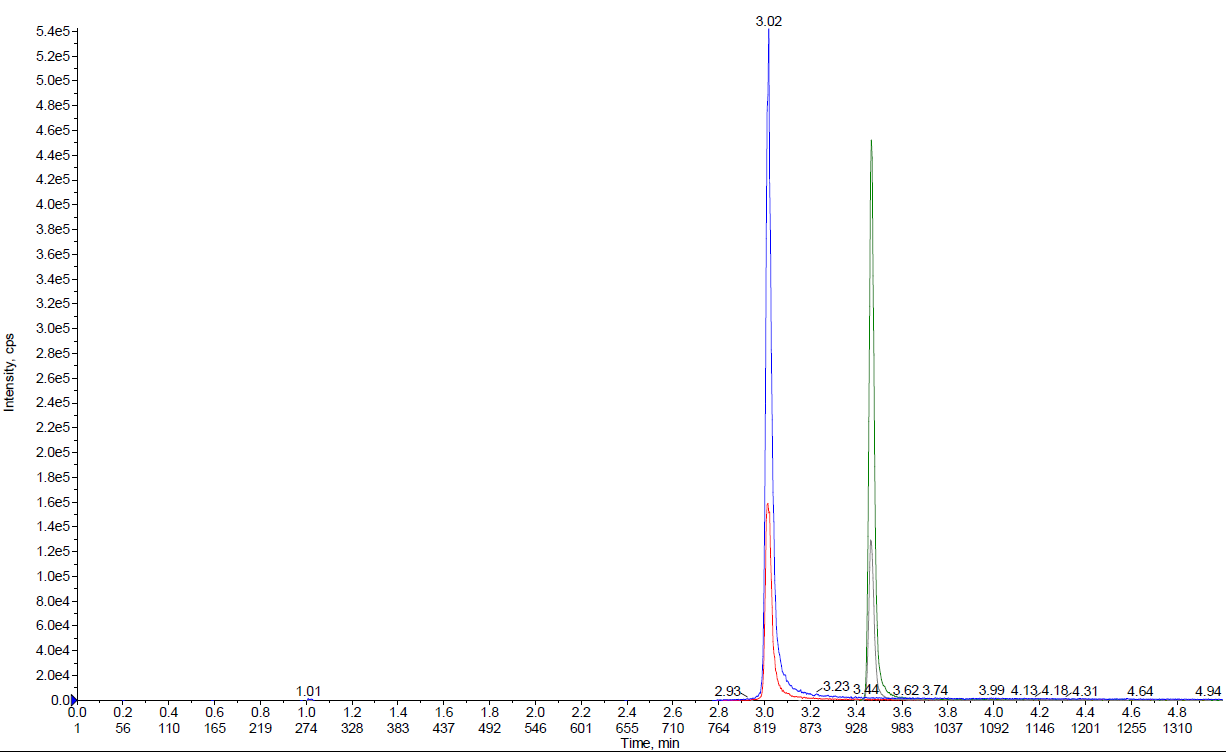
*

*Supplemental Figure 2: Example of a chromatogram of the 45-minute timepoint spiked with 4000 ng/mL. The blue peak represents the quantifier ion of methylene blue, the red peak the qualifier ion of methylene blue, the light green peak represents the quantifier ion of malachite green, and the dark green peak the qualifier ion of malachite green.*

- 1. Mass spectrometry

*Supplemental Table 1: Retention time (RT), multiple reaction monitoring transitions and compound-specific mass spectrometer settings. DP = declustering potential; EP = entrance potential; CE = collision energy; CXP = collision cell exit potential.*

| Compound | RT (min) | Precurser ion (m/z) | Product ion (m/z) | DP (V) | EP (V) | CE (V) | CXP (V) |
| --- | --- | --- | --- | --- | --- | --- | --- |
| Methylene blue quantifier | 3.02 | 283.9 | 252.1 | 100 | 10 | 67 | 14 |
| Methylene blue qualifier | 3.02 | 283.9 | 268.1 | 100 | 10 | 45 | 16 |
| Malachite green quantifier | 3.47 | 329.0 | 208.0 | 100 | 10 | 47 | 12 |
| Malachite green qualifier | 3.47 | 329.0 | 313.0 | 100 | 10 | 49 | 18 |

*Supplemental Table 2:* *System-specific parameters of the mass spectrometer.*

| Parameter | Value |
| --- | --- |
| Source temperature (°C) | 500 |
| IonSpray Voltage (V) | 5500 |
| Ion source gas 1 (psi) | 30 |
| Ion source gas 2 (psi) | 30 |
| Curtain gas (psi) | 35 |

1. Pharmacokinetic analysis

The concentration at each timepoint was determined by plotting the spiked concentrations against the ratio of the peak area of MB to the peak area of malachite green (*cfr.* internal standard). To fit the calibration curve, the aliquots spiked with 3000 ng/mL and 12000 ng/mL were excluded (*cfr.* accuracy), resulting in a total of four calibration points. The unknown concentration in the sample was quantified by determining the intercept on the x-axis. An illustration of the quantification process is provided in Suppl. Fig. 3.


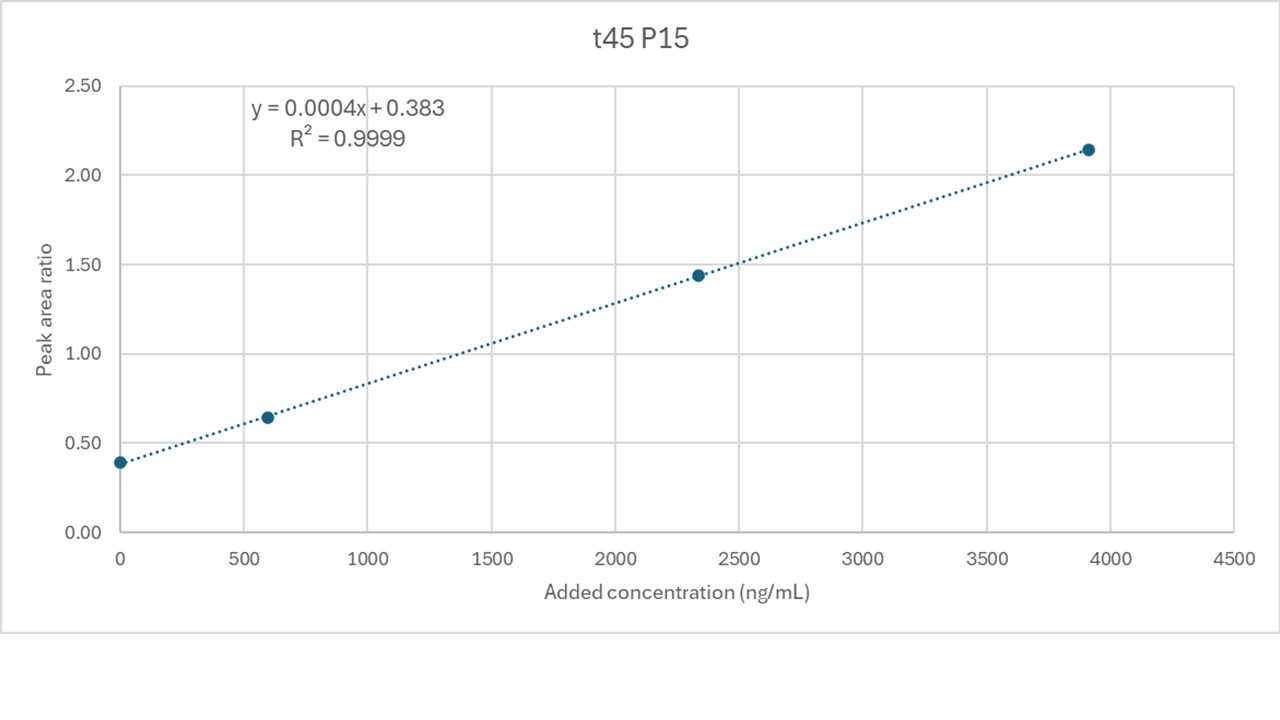


*Supplemental Figure 3: Illustration of applying the standard addition method to quantify the concentration of methylene blue present in a sample.*

*Supplemental Table 3: Absolute values of area under the curve (AUC) (corrected for individual weight) for each individual pig and corresponding values expressed relative to the AUC of reference pig 12. (P12).*

| Group | Individual pig number | Total AUC (ng*min/mL) | Compared to P12 (%) |
| --- | --- | --- | --- |
| 1 | P1 | 58082 | 72 |
|  | P12 | 80569 | 100 |
| 2 | P2 | 65024 | 81 |
|  | P4 | 6956520 | 70 |
| 3 | P3 | 7055934 | 69 |
|  | P5 | 70821 | 88 |
|  | P6 | 67199 | 83 |
| 4 | P7 | 57714 | 72 |
|  | P8 | 45302 | 56 |
| 5 | P9 | 48897 | 61 |
|  | P10 | 74769 | 93 |
| 6 | P14 | 84100 | 104 |
|  | P15 | 45850 | 57 |
